# Loss of submerged macrophytes in shallow lakes alters bacterial and archaeal community structures, and reduces their co-occurrence networks connectivity and complexity

**DOI:** 10.3389/fmicb.2024.1380805

**Published:** 2024-03-26

**Authors:** Jiahui Liu, Xianfei Huang, Xin Jiang, Chun Qing, Yue Li, Pinhua Xia

**Affiliations:** ^1^Guizhou Province Key Laboratory for Information System of Mountainous Areas and Protection of Ecological Environment, Guizhou Normal University, Guiyang, China; ^2^Guizhou Caohai National Nature Reserve Management Committee, Bijie, Guizhou, China

**Keywords:** bacterial community, archaeal community, sediment, water, loss of submerged macrophytes

## Abstract

**Introduction:**

Bacteria and archaea are important components in shallow lake ecosystems and are crucial for biogeochemical cycling. While the submerged macrophyte loss is widespread in shallow lakes, the effect on the bacteria and archaea in the sediment and water is not yet widely understood.

**Methods:**

In this study, 16S rRNA gene sequencing was used to explore the bacteria and archaea in samples taken from the sediment and water in the submerged macrophyte abundant (MA) and submerged macrophyte loss (ML) areas of Caohai Lake, Guizhou, China.

**Results:**

The results showed that the dominant bacterial phyla were *Proteobacteria* and *Chloroflexi* in the sediment; the dominant phyla were *Proteobacteria*, *Actinobacteriota*, and *Bacteroidota* in the water. The dominant archaea in sediment and water were the same, in the order of *Crenarchaeota*, *Thermoplasmatota*, and *Halobacterota*. Non-metric multidimensional scaling (NMDS) analyses showed that bacterial and archaeal community structures in the water were significantly affected by the loss of submerged macrophytes, but not by significant changes in the sediment. This suggests that the loss of submerged macrophytes has a stronger effect on the bacterial and archaeal community structures in water than in sediment. Furthermore, plant biomass (PB) was the key factor significantly influencing the bacterial community structure in water, while total nitrogen (TN) was the main factor significantly influencing the archaeal community structure in water. The loss of submerged macrophytes did not significantly affect the alpha diversity of the bacterial and archaeal communities in either the sediment or water. Based on network analyses, we found that the loss of submerged macrophytes reduced the connectivity and complexity of bacterial patterns in sediment and water. For archaea, network associations were stronger for MA network than for ML network in sediment, but network complexity for archaea in water was not significantly different between the two areas.

**Discussion:**

This study assesses the impacts of submerged macrophyte loss on bacteria and archaea in lakes from microbial perspective, which can help to provide further theoretical basis for microbiological research and submerged macrophytes restoration in shallow lakes.

## 1 Introduction

Shallow lake ecosystems are rich in bacteria and archaea community, which play a crucial role as decomposers in biogeochemical cycles, biodegradation and biotransformation of pollutants (Reid et al., 2019; Williams and Ducklow, 2019). However, bacterial communities have been found to be susceptible to a wide range of environmental factors, including abiotic (e.g., organic matter, nitrogen, phosphorus, and temperature) and biotic factors (e.g., aquatic plant succession; Cheng et al., 2016; Li et al., 2019; Ma et al., 2023). Archaea are the main prokaryotes after bacteria (Spang et al., 2017), whose distribution and diversity can also be affected by different environmental factors (Gordon-Bradley et al., 2014).

The loss of submerged macrophytes has become a serious ecological problem in shallow lakes worldwide (Rozemeijer et al., 2021). Zhang et al. (2017) conducted a quantitative assessment of 155 lakes around the world and found that the rate of submerged macrophyte loss (in cover and area) after 2000 was 33.6% ± 59.8% per year, especially for lakes larger than 50 km^2^, and the decreasing trend in submerged macrophytes was the most pronounced among all aquatic vegetations. Furthermore, a survey of submerged macrophytes in 14 typical shallow lakes in China found that Submerged macrophytes have been gradually degraded since the early 20th century (Huang et al., 2021). Caohai Lake is the largest shallow lake in Guizhou, since July 2020, the local waters of submerged macrophytes began to decline in large areas, the clear water state gradually to the turbid water state development (Li et al., 2023). Submerged macrophytes are the major producers in lake ecosystems, providing habitats for aquatic life, maintaining biodiversity, and inhibiting algal growth (Wood et al., 2017; Amorim and Moura, 2020; Wang et al., 2021; Misteli et al., 2023). However, the gradual loss of submerged macrophytes can have certain impact on shallow lake ecosystems. It will not only by can trigger the redistribution of various nutrients in sediment and water (Huang et al., 2021), and even cause the lake to transform from a clear water state dominated by submerged macrophytes to a turbid water state dominated by phytoplankton, with a consequential decline in its ecological functions (Ardichvili et al., 2023).

Additionally, there is a close interrelationship between submerged macrophytes and microorganisms. Submerged macrophyte root systems can provide sites for microbial activities in sediment (Ma et al., 2023), promote microbial aerobic decomposition when the root system releases oxygen into the sediment (Wang et al., 2017), and macrophyte apoplasts enriched on the surface of sediment add nutrient sources for microorganisms (Liu et al., 2020; Chao et al., 2021). Thus, when submerged macrophytes growth are altered, the microbial community in the sediment may also change. Meanwhile, submerged macrophytes can directly influence microbial community through the release of chemosensory substances from their leaves (Chao et al., 2021). They can also alter conditions such as nutrient levels or pH value in the water to enhance environmental heterogeneity, thereby indirectly effecting the microbial composition and diversity in the water (Logue et al., 2012; Dai et al., 2019; Ren et al., 2023). Numerous studies have shown that the submerged macrophyte biomass has effect on the bacterial community composition and diversity in the sediment and the water (Zhao et al., 2013; Chao et al., 2021; Wu et al., 2021). However, a report had found that the presence or absence of submerged macrophytes has no significant effect on sediment bacteria communities (Liu W. Z. et al., 2018). Furthermore, Chao et al. (2021) found that the alpha diversity of bacteria in the water did not differ significantly between submerged macrophytes restored and submerged macrophytes bare areas. The influence of submerged macrophytes on the bacterial community composition and diversity are thus controversial in the previously published literature. Archaea has been found to have multiple functions in ammonia oxidation, methane metabolism, and organic matter degradation (Orphan et al., 2002; Könneke et al., 2005; Brochier-Armanet et al., 2008; Lloyd et al., 2013). Currently, most studies on archaea have focused on marine ecosystems (Liu et al., 2020; Li et al., 2021), while fewer studies have been conducted on archaea in lakes. Although a few studies have examined the archaeal community composition and diversity in lakes, the network patterns of archaea have not been analyzed (Haller et al., 2011; Fan and Xing, 2016; Zhang et al., 2020). In addition, there is a virtual void of research on the relationship between submerged macrophytes loss and archaea community in lakes.

Caohai Lake is a typical clear-water grass-type lake in China; however, in recent years its submerged macrophytes have been largely lost, and how this phenomenon affects the bacterial and archaeal community in the lake is not yet known. Therefore, we collected the water and sediment in submerged MA and submerged ML areas of Caohai Lake for the study. In this study, the 16S rRNA amplicon sequencing technology was used and combined with a variety of statistical analysis methods to analyze the bacterial and archaeal community composition, diversity, and their co-occurrence networks. We hypothesized that the loss of submerged macrophyte would affect bacterial and archaeal communities structure and co-occurrence network, so the aims of the study were (1) understanding differences in community composition and diversity of bacteria and archaea between MA and ML areas? Determine the main environmental factors that influence the structure of bacterial and archaeal communities; (2) determine whether the loss of submerged macrophytes has altered the co-occurrence network of bacterial or archaeal communities.

## 2 Materials and methods

### 2.1 Study area and field sampling

Caohai Lake (26°47′N–26°52′N, 104°9′E–104°20′E) is located in the southwest of Weining County in Guizhou Province, China. It is a typical shallow lake on the Yunnan-Guizhou Plateau, with a surface water area of 22.39 km^2^ and a mean depth of 2 m. Caohai Lake has a subtropical highland monsoon climate with an annual mean temperature of 10.9°C. The rainy season in the Caohai Lake is from May to October and the dry season is from December to March (the dry period), with an average annual rainfall of 950 mm. Aquatic plants flourish during the flood period, while many die during the dry period. Common submerged macrophytes included *Potamogeton perfoliatus* L., *Najas marina* L., *Myriophyllum spicatum* L., and *Potamogeton malaianus* Miq.

**Figure 1:**
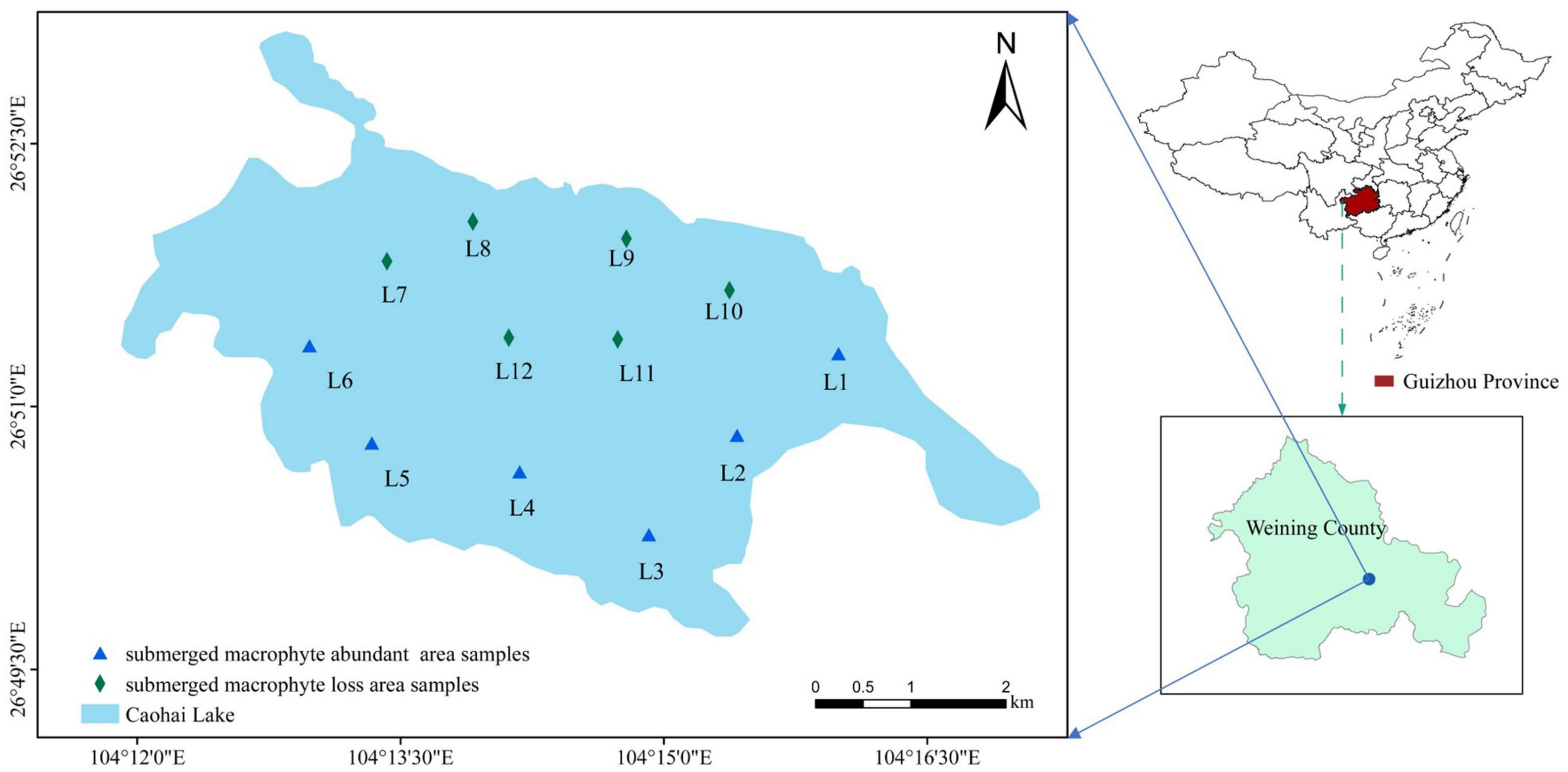
Locations of the sampling sites in Caohai Lake, Guizhou.

In October 2021, samples were collected from 6 sampling points (L1~L6) in the MA area (Figure 1), and the submerged plants in this area were *Potamogeton pectinatus* L., *Potamogeton malaianus* Miq., *Najas marina* L., *Myriophyllum spicatum* L., and *Nitella acuminate* B. Samples were collected from 6 sampling points (L7~L12) in the ML area (Figure 1), and the only submerged plants in this area were *Najas marina* L., and *Nitella acuminate* B. A polyethylene water collector was used to collect 3 L of the water at a depth of 0.5 m at each sampling point and pour it into a polyethylene bottle. Subsequently, dissolved oxygen (DO), pH, and Oxidation–Reduction Potential (ORP) were determined on-site using a portable water quality analyzer (HQ30d, HACH, United States). In addition, water transparency (WT) was measured using Secchi discs and water depth (WD) was measured using deep-water rods. At each sampling site, surface sediment (top 10 cm) was collected three times with a grab sampler, mixed well, and then approximately 15 g of the mixed sample was placed in a centrifuge tube and kept in a holding tank for low temperature maintenance. Water and sediment samples collected were sent to the laboratory within 12 h for cryopreservation or pretreatment. Around each sampling point, 3 to 5 sample plots (1 m^2^) were set to investigate the surrounding submerged macrophytes. A homemade underwater sickle (25 × 25 cm) was used to collect submerged macrophytes in per sample plot. The collected submerged macrophytes were cleaned, toweled to absorb water and then weighed to calculate the PB (g/m^2^). The plant coverage (PC%) was calculated based on observations at the sampling sites, and the formula is as follows:


$$PC=\frac{\begin{array}{l} \mathrm{Total area covered}\;by\;\mathrm{submerged}\\ \mathrm{macrophytes in the sample plots}\;\left(㎡\right) \end{array}}{\mathrm{Total sample plot areas}\;\left(㎡\right)}\times 100\%$$

### 2.2 Laboratory analysis

Water and sediment samples were sent to the laboratory, the sediment samples were stored in a refrigerator at −20°C for 16S rRNA amplicon sequencing. The water samples were divided into two subsamples, one for 16S rRNA amplicon sequencing and the other for physicochemical analysis. Bacteria and archaea in the water were collected by taking 500 mL of water samples through a 0.22 μm polycarbonate membrane, and then the membrane was stored in a refrigerator at −20°C until DNA extraction. Determination of TN and total phosphorus (TP) requires digestion of water samples with potassium persulfate, followed by determination by Ultraviolet–visible spectrophotometry (Liu Z. W. et al., 2018). The water samples were filtered through 0.45 μm Millipore membrane, then the ammonia nitrogen (NH_3_-N) is determined according to the Nessler’s reagent spectrophotometry method (Bai et al., 2020). Chlorophyll *a* (Chl *a*) was spectrophotometrically determined after extraction with 90% ethanol (Te et al., 2017). The concentration of chemical oxygen demand (COD_Mn_) in water was determined under acidic and boiling water bath heating conditions according to standard methods (Dai et al., 2019).

### 2.3 DNA extraction, amplification, and sequencing

DNA was extracted from water and sediment samples according to the instructions of the E.Z.N.A.® soil DNA Kit (Omega Bio-tek, Norcross, GA, United States). The extracted DNA concentration and purity were measured using NanoDropTM 2000 (Thermo Fisher Scientific, United States) and DNA quality was measured using 1% agarose gel electrophoresis, the qualified DNA was then stored at −20°C until further analysis. The V3-V4 region of the bacteria was amplified using the primers 338F (ACTCCTACGGGAGGCAGCAG) and 806R (GGACTACHVGGGTWTCTAAT; Chao et al., 2021). The V3-V4 region of archaea was amplified using the primers 524F10extF (TGYCAGCCGCCGCGGTAA) and 958R (YCCGGCGTTGAVTCCAATT; Chen and Wen, 2021). The gene amplification was performed in a 20 μL reaction system containing 4 L of FastPfu buffer (5 ×), 2 μL of dNTP mixture (2.5 mM), 0.8 μL of each primer (5 μM), 0.4 μL of Fastpfu polymerase, and 10 ng of template DNA. The PCR amplification parameters were as follows: initial denaturation at 95°C for 3 min, followed by 27 cycles of denaturation at 95°C for 30 s, 55°C for 30 s, and then extension at 72°C for 30 s, with a final extension at 72°C for 10 min. PCR products were recovered using a 2% agarose gel, and product purification was carried out using the DNA Gel Recovery Purification Kit (PCR Clean-Up Kit, Passover, China). The purified PCR product was then subjected to library construction using the NEXTFLEX Rapid DNA-Seq Kit.

PCR sequencing was performed using the Illumina PE300/PE250 platform (Illumina, San Diego, United States) according to the standard protocols of Majorbio Bio-Pharm Technology Co. Ltd. (Shanghai, China). After sample splitting of PE reads obtained from Illumina sequencing, the optimized data were firstly obtained by quality control and filtering of double-ended Reads based on sequencing quality, and also splicing based on the overlap relationship between double-ended Reads. The optimized data were then processed using sequence noise reduction methods (DADA2/Deblur, etc.) to obtain ASV (Amplicon Sequence Variant) information. In order to minimize the impact of sequencing on data analysis, the number of sequences of all samples was flatten, and the average sequence coverage of each sample after flatting was 99.1%.

### 2.4 Data analyses

A sampling map was created using ArcGIS (version 10.8). An independent sample t-test was used to compare the differences in water quality and submerged macrophyte characteristics between the MA and ML areas [data were transformed using $\log\left(x+1\right)$ conversion to enhance their normality]. The Wilcoxon test (non-parametric) was used to compare the differences in the alpha diversity of the bacterial and archaeal communities between the MA and ML areas on IBM SPSS Statistics (Version 23.0; Philonenko and Postovalov, 2015). The NMDS based on the Bray-Curtis distance were implemented using the R (version 4.0) packages “vegan” and “ggplot2” to reveal the differences in the bacterial and archaeal community compositions between the two areas (Wan et al., 2021). The “vegan”R package was used to calculate variance inflation factor (VIF) and the forward selection principle was used to exclude environmental factors with high correlation (excluding variables with VIF > 10). The redundancy analysis (RDA) tests were performed using the “vegan” R package to determine the effects of environmental factors on the bacterial and archaeal community structures in water (Chao et al., 2021). Network analyses are used to reveal species interactions in bacterial and archaeal community in the sediment and the water. In order to improve the reliability of the network analysis, Amplicon Sequence Variants (ASVs) with the top 200 abundances were selected to calculate pairwise Spearman correlations. Dominant ASVs with Spearman’s correlation coefficient (*ρ*) > 0.6 and false discovery rate-corrected (FDR-corrected) *p*-value <0.01 were screened. This was then visualized using the Gephi (version 0.9.2) platform. In order to better evaluate the networks pattern affected by the loss of submerged macrophytes, we used Gephi (version 0.9.2) to calculate the network topology parameters (Nodes, Edges, Modularity, Average degree, Average clustering coefficient, and Average path length).

## 3 Results

### 3.1 Variations in the bacterial and archaeal community composition and diversity between the MA and ML areas

**Figure 2:**
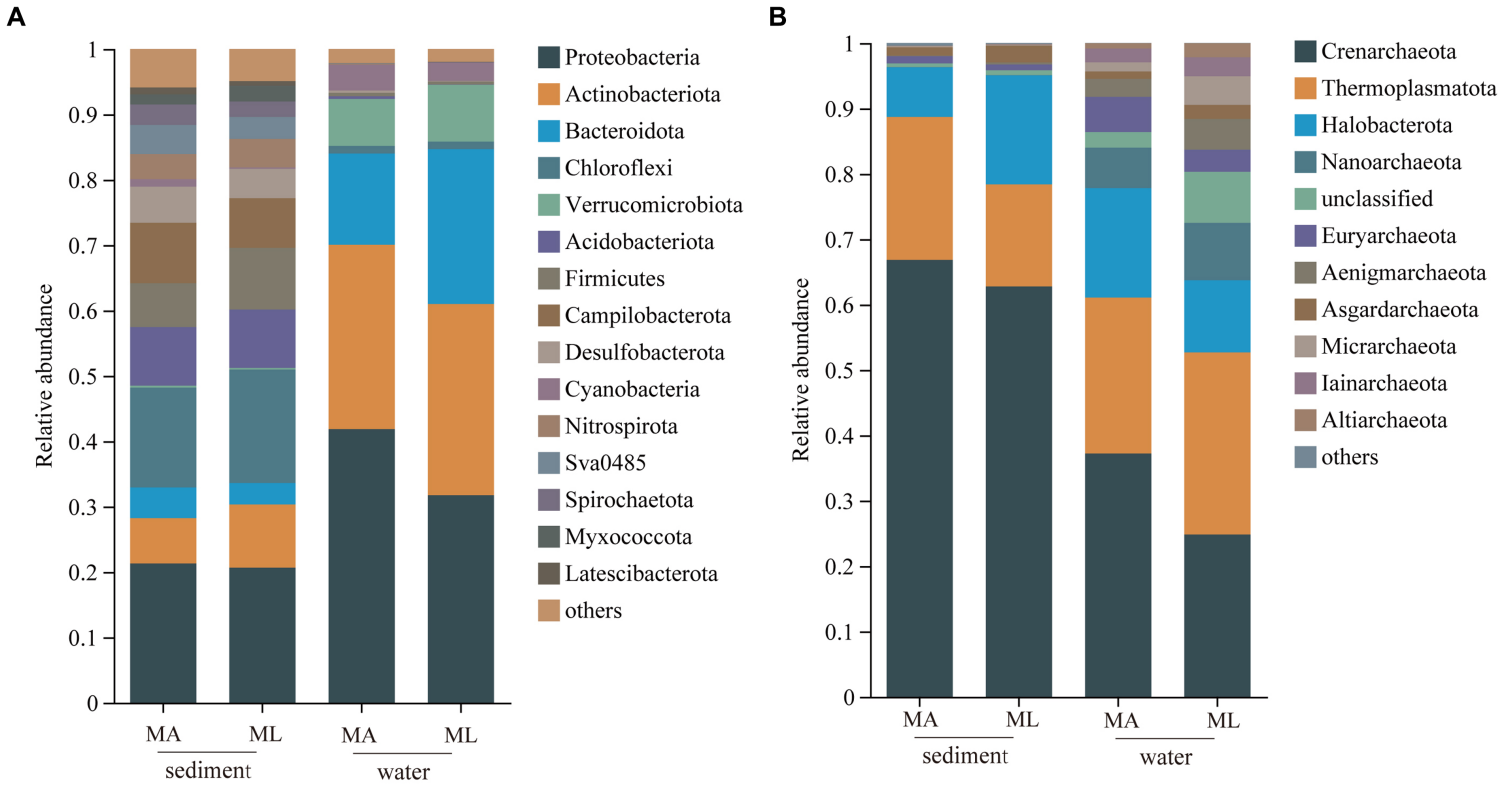
At the phylum level, the average relative abundance of the bacteria **(A)** and archaea **(B)** in sediment and water in the submerged macrophyte abundant (MA) area and submerged macrophyte loss (ML) area.

**Figure 3:**
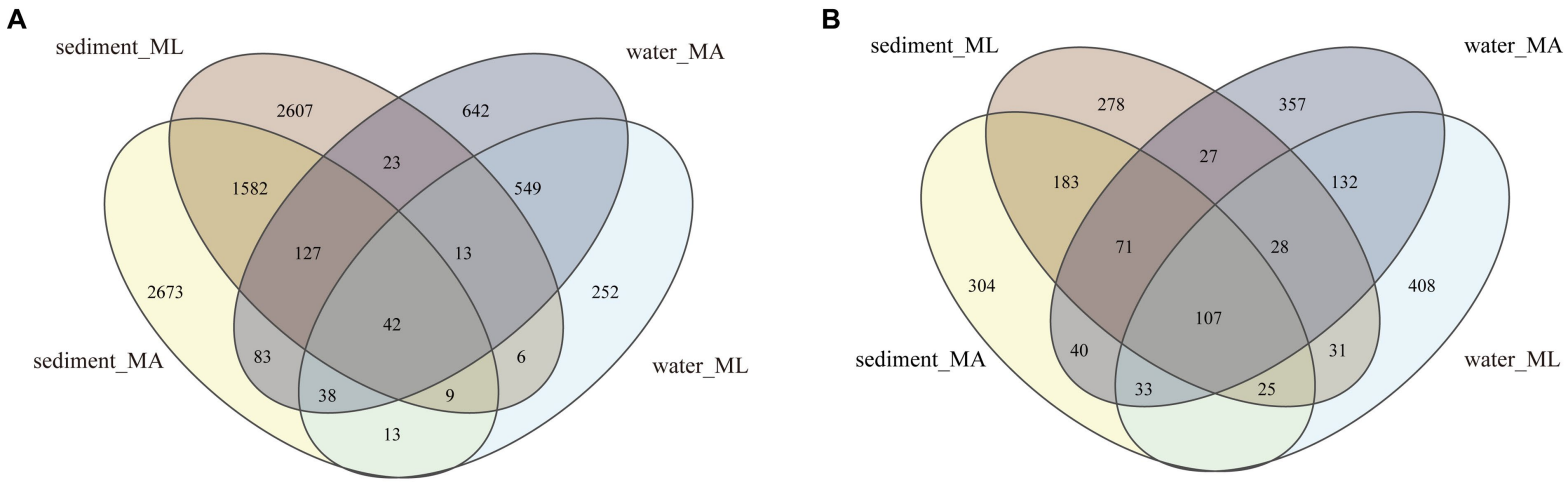
At the ASV level, Venn diagrams of bacteria **(A)** and archaea **(B)** community in sediment and water in the submerged macrophyte abundant (MA) area and submerged macrophyte loss (ML) area.

In the sediment, the dominant bacterial phyla were *Proteobacteria* and *Chloroflexi*. The relative abundance of *Proteobacteria* in the MA and ML areas was similarity, which were 21.3% and 20.7%, respectively; the relative abundance of *Chloroflexi* was 15.3% in the MA area and 17.4% in the ML area; (Figure 2A). 1,760 ASVs were shared between the MA and ML areas, and each area had 2,807 and 2,649 unique ASVs, respectively (Figure 3A). In the water samples, *Proteobacteria*, *Actinobacteriota*, and *Bacteroidota* were the dominant bacteria, which had relative abundance of 41.9%, 28.2%, and 14.0% in the MA area, and 31.8%, 29.3%, and 23.7% in the ML area, respectively (Figure 2A). Moreover, two areas shared 642 ASVs, unique ASVs was higher in the MA area than that in the ML area (875 and 280, respectively; Figure 3A).

The dominant archaeal phyla were *Crenarchaeota*, *Thermoplasmatota,* and *Halobacterota* in the sediment and the water samples. In the sediment samples, relative abundances of *Crenarchaeota* and *Thermoplasmatota* in MA area were slightly higher than those in ML area, and their relative abundances in the MA area were 66.8% and 21.9%, and 62.8% and 15.6% in the ML area (Figure 2B). However, the relative abundance of the *Halobacterot* was lower in the MA area than that in the ML area (7.6% and 16.7%, respectively; Figure 2B). We observed that there were 386 ASVs shared between MA and ML areas, and the number of unique ASVs in MA and ML areas was close (401 and 364, respectively; Figure 3B). In water, compared with the MA area, the relative abundance of *Crenarchaeota* (MA:37.2%; ML: 24.8%) and *Halobacterota* (MA: 16.8%; ML: 11.0%) was lower in the ML area (Figure 2B). However, the relative abundance of the *Thermoplasmatota* in the MA area (23.9%) was lower than that in the ML area (27.9%; Figure 2B). 300 ASVs were shared between the MA and ML areas, while the number of unique ASVs did not differ much between the two areas (495 and 488, respectively; Figure 3B).

**Figure 4:**
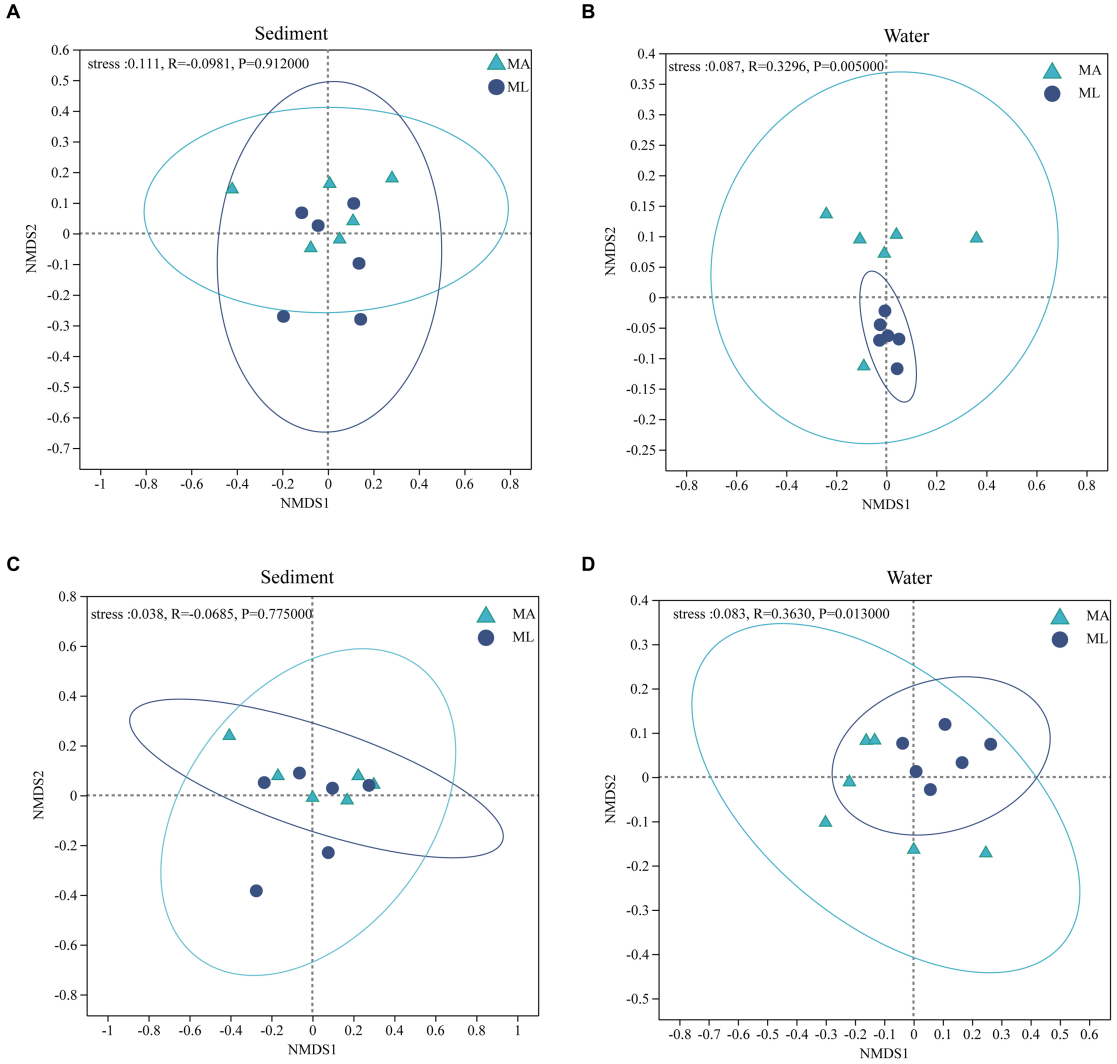
At the genus level, non-metric multidimensional scaling (NMDS) ordination of the bacteria **(A,B)** and archaea **(C,D)** community in sediment and water between the submerged macrophyte abundant (MA) area and submerged macrophyte loss (ML) area (calculated using Bray-Curtis).

According to the results of the NMDS analysis, the bacterial and archaeal community structures in the sediment were not significantly different between the MA and ML areas (*p* > 0.05; Figures 4A,C). However, the bacteria community in water was significantly segregated between the MA and ML, and the results was confirmed by the ANOSIM test (R = 0.3296, *p* < 0.01; Figure 4B). Moreover, there was significant differences in the archaeal community structure in water between MA and ML (R = 0.3630, *p* < 0.05; Figure 4D). To obtain information on the richness and diversity of bacterial and archaeal communities, we have calculated alpha diversity indices (Shannon, Simpson, Chao1, and PD). The results showed that the bacterial alpha diversity in both water and sediment did not change significantly between MA and ML areas (Supplementary Table S1). However, Chao1 of in water bacteria was slightly higher in the MA than in the ML. For archaea in the sediment, Shannon, Simpson, Chao1, and PD were similar for MA and ML areas (Supplementary Table S1). It is worth mentioning that although there was no significant difference in the diversity indices (Shannon and Simpson) of archaea in the water, Chao1 and PD were higher in the ML area.

### 3.2 Relationship between environmental factors and the water bacterial and archaeal community structures

The environmental factors, including TN, TP, NH_3_-N, COD_Mn_, Chl *a*, pH, and ORP, were not significantly different between the MA and ML areas (*p* > 0.05). However, the TN, TP, COD_Mn_, pH, and NH_3_-N contents in the MA area were slightly higher than those in the ML area (Supplementary Table S2). The DO, WD, and WT/WD were significantly different between the two areas (*p* < 0.05), and the DO content and WT/WD were significantly higher in the MA area than in the ML area, whereas the WD showed the opposite change (Supplementary Table S2). The PB and PC were significantly higher in the MA area than in the ML area (Supplementary Table S2).

**Figure 5:**
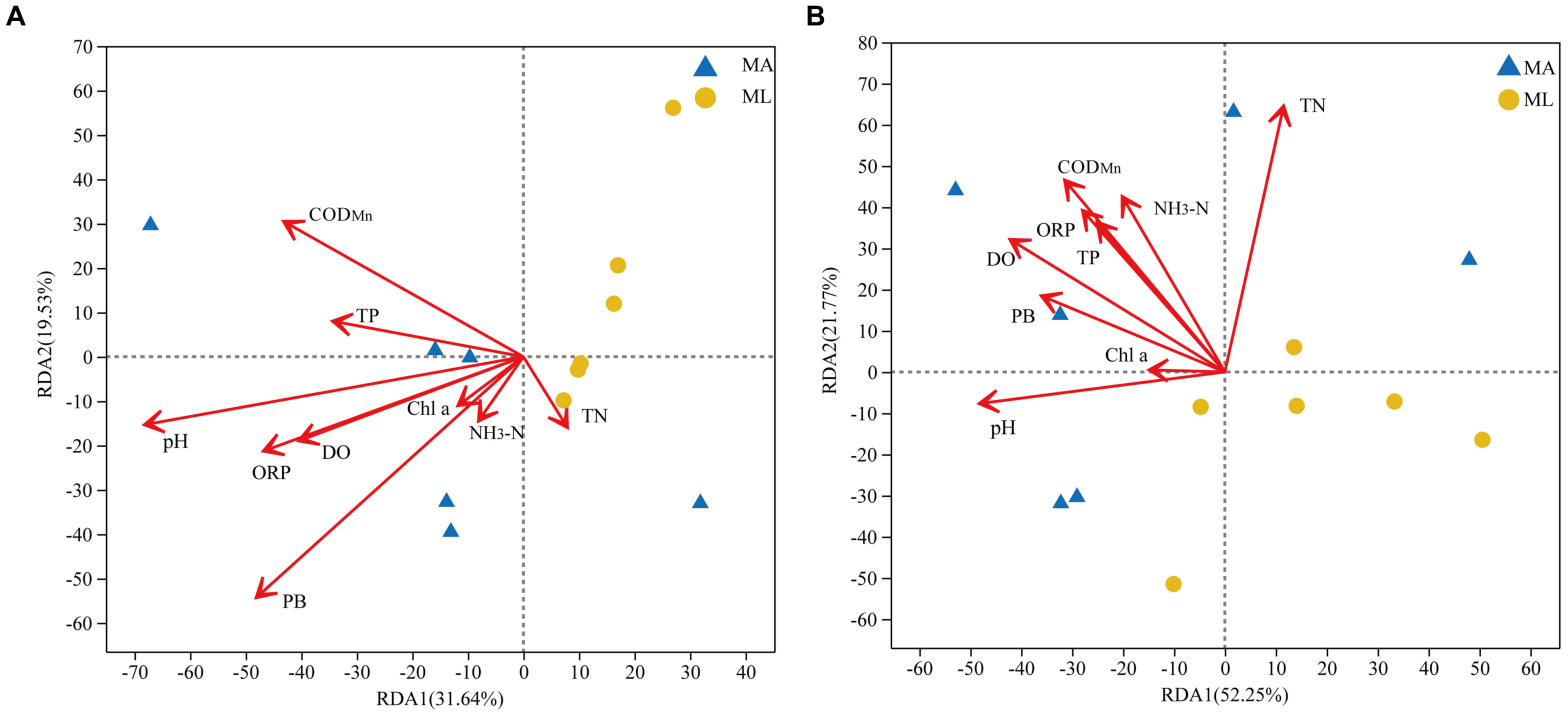
Redundancy analysis (RDA) of the bacterial **(A)** and archaeal **(B)** community structures in water and environmental factors between the submerged macrophyte abundant (MA) area and the submerged macrophyte loss (ML) area.

RDA analyses were performed to clarify the association between bacterial and archaeal community structures and environmental factors. The environmental factors were screened using the forward selection principle, and the PC was finally eliminated. All selected factors on both axes explained 51.2% of the changes in the water bacterial community structure (Figure 5A) and 74.0% of the changes in the water archaeal community structure (Figure 5B). The RDA results showed that PB (R^2^ = 0.5209, *p* < 0.05) and pH (R^2^ = 0.5, *p* < 0.05) were important factors that significantly affected the bacterial community structure in the water, followed by COD_Mn_, DO, and ORP, which were also environmental variables explaining the changes in the bacterial community structure (Figure 5A). Furthermore, TN (R^2^ = 0.5114, *p* < 0.05), pH, DO, NH_3_-N, and COD_Mn_ were identified as important factors affecting the archaeal community structure in the water, among which TN was the most significant (Figure 5B).

### 3.3 Network patterns of the bacteria and archaea show change with loss of submerged macrophytes

**Figure 6:**
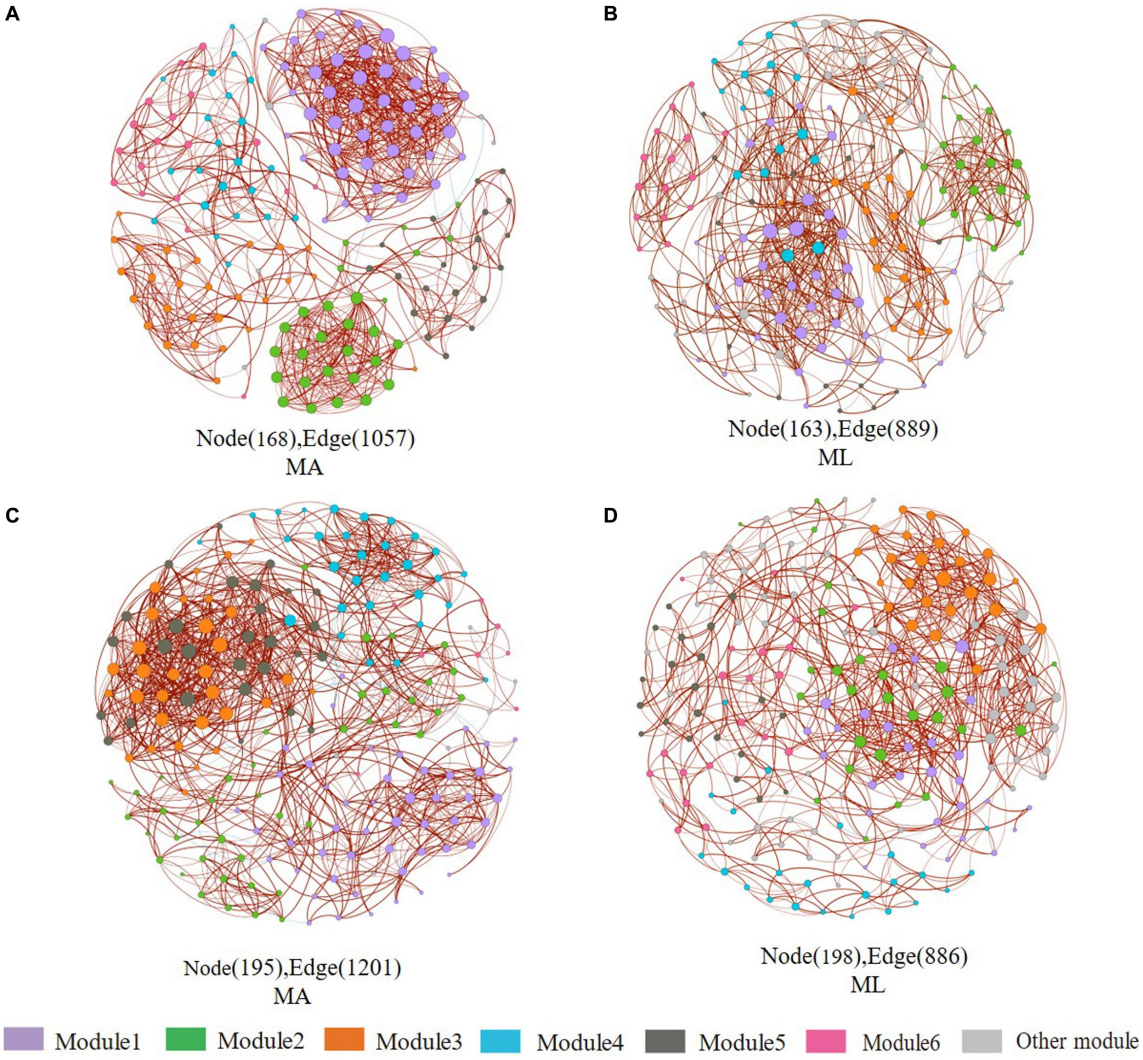
Co-occurrence networks of the sediment and water bacteria community in the submerged macrophyte abundant area (MA) and the submerged macrophyte loss (ML) area. The size of each node is proportional to the number of connections (i.e., degrees) and they are classed according to the modularity (aggregation of similar functions into one module). The red and blue edges (lines) in the network indicate significant positive and negative correlations, respectively. Sediment bacterial networks in the MA **(A)** area and ML **(B)** area; water bacterial networks in the MA **(C)** area and ML **(D)** area.

**Figure 7:**
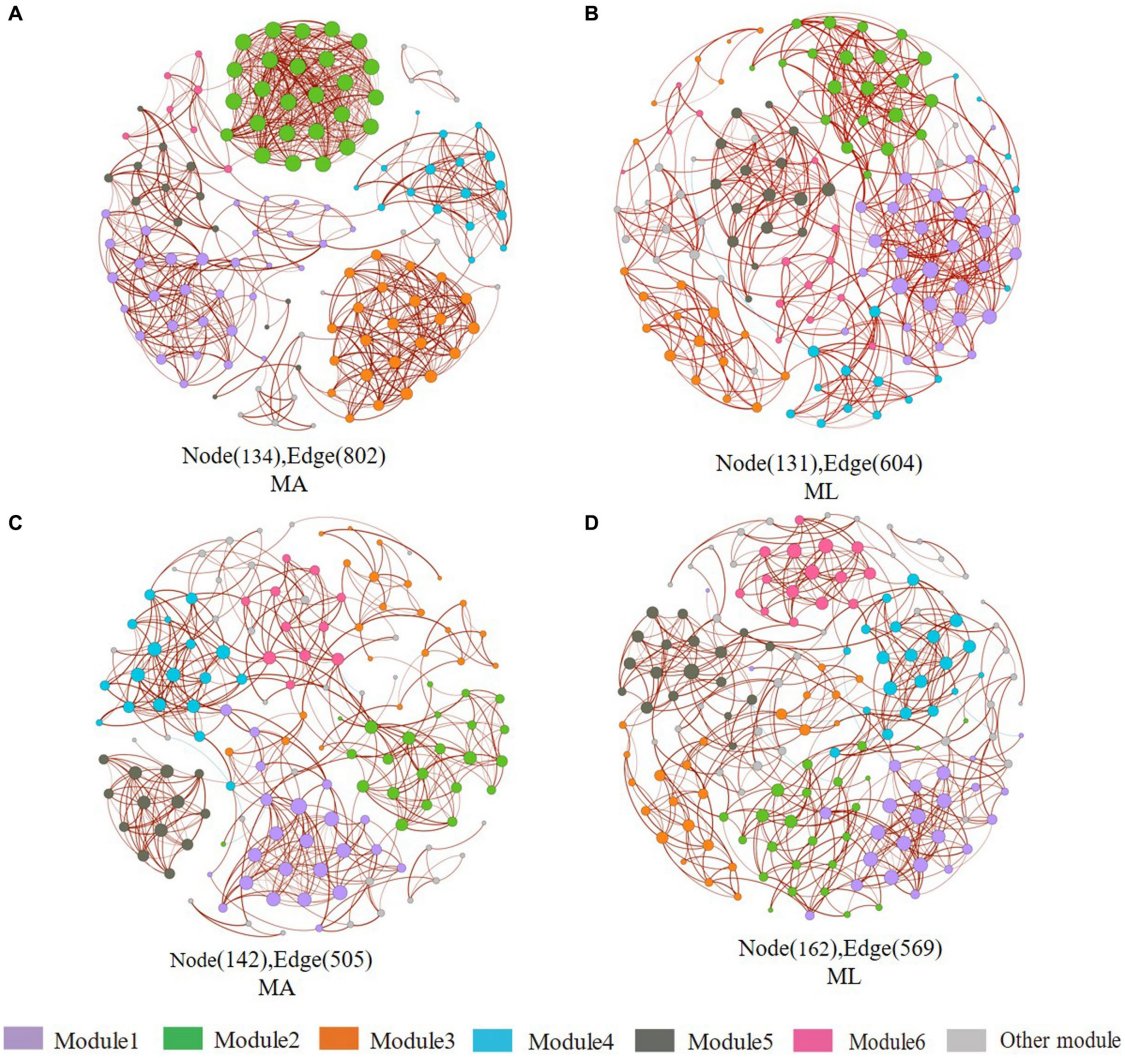
Co-occurrence networks of the sediment and water archaea community in the submerged macrophyte abundant (MA) area and the submerged macrophyte loss (ML) area. The size of each node is proportional to the number of connections (i.e., degrees) and they are classed according to the modularity (aggregation of similar functions into one module). The red and blue edges (lines) in the network indicate significant positive and negative correlations, respectively. Sediment archaea networks in the MA **(A)** area and ML **(B)** area. Water archaea networks in the MA **(C)** area and ML **(D)** area.

According to the different genotypes and mediators, eight co-occurrence networks were constructed for the MA and ML areas to reveal the changes of the submerged macrophyte loss on the bacterial and archaeal community co-occurrence networks (Figures 6, 7). All co-occurrence networks were divided into 6 main modules and tend to co-occur among ASVs (positive correlation; Figures 6, 7). In the sediment bacterial network, the nodes and edges of the MA network are 168 and 1,057, respectively, and the nodes and edges of the ML network are 163 and 889 (Figures 6A,B), and the MA and ML network nodes were mainly occupied by *Proteobacteria* and *Chloroflexi* (Supplementary Figures S1A,B). The water MA network consists of 195 nodes and 1,202, and 198 nodes and 886 edges for the water ML, indicating that the connectivity was greater in MA network (Figures 6C,D). *Proteobacteria*, *Bacteroidota*, and *Actinobacteriota* accounted for 83.1% of the nodes in the water MA network, and the module 1 consisted mainly of *Proteobacteria* (Supplementary Figure S1C), whereas *Proteobacteria* was more dispersed in the water MA network (Supplementary Figure S1D). In addition, the average degree and average clustering coefficient were higher for MA than for ML in the water and sediment bacteria networks, and the average path length was larger for ML (Table 1).

**Table 1:** Topological indices for the associations of the co-occurrence networks for the bacterial and archaeal community in the submerged macrophyte abundant (MA) and submerged macrophyte loss (ML) areas.

			Nodes	Edges	Modularity	Average clustering coefficient	Average degree	Average path length
Bacteria	Sediment	MA	168	1,057	0.680	0.597	12.583	2.62
		ML	163	889	0.647	0.562	10.908	3.881
	Water	MA	195	1,201	0.639	0.555	12.318	3.942
		ML	198	886	0.627	0.487	8.949	4.221
Archaea	Sediment	MA	134	802	0.767	0.941	11.97	7.767
		ML	131	604	0.722	0.595	9.221	4.444
	Water	MA	142	505	0.755	0.568	7.113	5.003
		ML	162	569	0.772	0.511	7.025	4.744

In sediment archaea networks, the MA network consists of 134 nodes and 802 edges, and 131 nodes and 604 edges for the ML network (Figures 7A,B). Additionally, the MA network average degree was 11.97, and the average clustering coefficient was 0.941; the ML network average degree was 9.221, and the average clustering coefficient was 0.595 (Table 1). Moreover, *Crenarchaeota* in the MA network clustered in module 2 (Supplementary Figure S2A), but *Crenarchaeota* in the ML network clustered in module 1 (Supplementary Figure S2B). In water archaea networks, we observed no significant change in the MA and ML network patterns, with little fluctuation in the number of nodes (MA: 142, ML: 162), edges (MA: 505, ML: 569), average degree, average clustering coefficient and average path length (Figures 7C,D; Table 1), suggesting that the archaeal networks structure in water were not significantly affected by submerged macrophyte loss. *Crenarchaeota*, the most dominant archaea, occupied 41.4% nodes in the water MA network and 28.4% in the water ML network and was dispersedly distributed across modules (Supplementary Figures S2C,D).

## 4 Discussion

### 4.1 Effects of submerged macrophyte loss on the composition and diversity of bacterial and archaeal communities

The results showed that the loss of submerged macrophytes had effect on the bacterial and archaeal community compositions in the sediment and water. The dominant bacterial phyla in water and sediment samples were similar in the MA and ML areas, but there was difference in the relative abundance of the dominant bacterial phyla (Figure 2A), the result that was consistent with the results of a study (Chao et al., 2021). We observed that the relative abundance of *Proteobacteria* in sediment fluctuated little between the MA and ML areas, but the relative abundance of *Chloroflexi* differed between the two areas. Since *Chloroflexi* has extremely diverse nutritional modes, the nutritional modes and metabolic pathways of *Chloroflexi* change with environmental change (Xian et al., 2020). Therefore, there are differences in the distribution of *Chloroflexi* in the sediment of the two areas with different submerged macrophytes biomass and species. In the water, *Proteobacteria* were enriched in the MA area, and on the contrary, *Bacteroidota* were significantly enriched in the ML area. Changes in *Bacteroidota* may be due to loss of submerged macrophytes increasing the concentration of dissolved organic matter (DOC) in the water (Van der Gucht et al., 2005), which promotes the colonization of *Bacteroidota* (Dai et al., 2019). In addition, we analyzed the changes of unique species between different areas and found that the number of unique species in sediment and water decreased with the loss of submerged macrophytes (Figure 3A), which we hypothesized might be related to the biomass and species of the submerged macrophytes. When the biomass and species of submerged macrophytes decreased, the active selection ability of macrophytes for some unique bacteria was weakened (Bulgarelli et al., 2013), resulting in fewer unique species in ML area.

*Crenarchaeota* and *Thermoplasmatota* are the most widely distributed species in the lake (Fillol et al., 2015; Zhang et al., 2020). Our results also indicate that *Crenarchaeota* and *Thermoplasmatota* were the dominant archaea in water and sediment samples. Furthermore, we found that the loss of submerged macrophytes led to a decrease in the relative abundance of *Crenarchaeota* in sediment and water. This change may be due to lignin, which was a major component of the macrophyte cell wall that promote growth of *Crenarchaeota* (Yao et al., 2015). The lower biomass of submerged macroplants in the ML area reduced the amount of lignin diffused into water and sediment, thereby inhibiting *Crenarchaeota* enrichment (Yu et al., 2018). However, there were difference in *Thermoplasmatota* enrichment in sediment and water. *Thermoplasmatota* in sediment is enriched in the MA area, while *Thermoplasmatota* in water are enriched in ML area, and this difference is attributed to the different responses of species in different media to the external environment (Liu et al., 2020).

According to NMDS analysis the loss of submerged macrophytes had significant effect on the bacterial and archaeal community structures in the water (Figures 4B,D), while there was no significant effect on the bacterial and archaeal communities in the sediment (Figures 4A,C). This may be due to the fact that the bacteria community structures in water is more sensitive to environmental change and more susceptible to external environmental disturbances (Zeng et al., 2019; Jiao et al., 2020). A number of studies have shown that PB, PC, and plant species are key factors that directly affect bacterial structure in water (Dai et al., 2019; Ma et al., 2020; Wu et al., 2021), and our result also confirmed that PB is the most dominant key variable (Figure 5A). In general, submerged macrophytes could produce a wide range of fresh substrates such as ions, free oxygen, and a variety of primary and secondary metabolites (Uren, 2007; Haichar et al., 2014). As the submerged macrophytes dies the types and content of substrates acting on bacteria and archaea change, with a subsequent increase in microbial heterogeneity between the two areas, leading to changes in microbial community structure. In addition, water nutrients as common source of competition between microorganisms and submerged macrophytes (Soana and Bartoli, 2014). Since PB and PC differ significantly between MA and ML areas (Supplementary Table S2), microbial in MA and ML areas have different competitive pressures on nutrient sources, which contributes to increase microbial heterogeneity altering their community structure. However, the key environmental factors affecting archaeal community structure were different from those of bacteria. According to the RDA analysis, the change in the archaeal community structure in the water were mainly caused by TN, pH, DO and NH_3_-N, with TN being the key factor (Figure 5). It might be due to the significant nitrogen metabolism and greater nitrogen fixation potential of archaea compared to bacteria, which require more TN content for these processes (Zhang et al., 2020). In addition, different competition for TN by submerged macrophytes in the MA and ML areas resulted in different TN available to the archaea in the two areas, thus it is reasonable for TN to be a key factor limiting changes in archaeal community structure. The number of submerged macrophytes was a controlling factor for the whole lake area, and submerged macrophytes can change the surrounding water quality conditions (Dai et al., 2019; Wu et al., 2021). We believed that changes in archaeal community structure may result indirectly from significant differences in DO levels in water. This is due to the presence of key enzymes with oxygen metabolism in *Crenarchaeota* that catalyze the reduction of oxygen to water (Vetriani, 2001), and thus differences in DO drive structural changes in archaea. The effect of DO on the archaeal community structure in water has also been found in study on the Jiulong River (Tang, 2022). The loss of submerged macrophytes had no significant effect on the alpha diversity of bacterial and archaeal community in water and sediment (Supplementary Table S1). Although excess organic matter produced by loss of submerged macrophytes promoted the growth of heterotrophic microorganisms in sediment, it also hindered the growth of facultative and obligate microorganisms (Remmas et al., 2016; Wu et al., 2017), thus the diversity of bacteria and archaea in sediment did not change significantly. In addition, the alpha diversity of microorganisms in water mainly depends on the availability of nutrients in the water, and our results have shown that the differences in nutrient conditions (e.g., TN and TP) in the two areas were not significant, and therefore the changes in alpha diversity were not significant (Logue et al., 2012; Chao et al., 2021).

### 4.2 Responses of bacterial and archaeal community network patterns to the submerged macrophyte loss

Complex interactions and co-occurrence patterns exist between microbial communities (Jiao et al., 2020), and co-occurrence networks can reveal complex interactions of microbial communities (Stenuit and Agathos, 2015). In this study, we found that the complexity and connectivity of water and sediment bacteria networks were weaker in the ML area (Figure 6). The connectivity and complexity of sediment archaea network were strong in the MA area, while the water archaeal networks were nonsignificant differences between the MA and ML areas (Figure 7). It can be seen that submerged macrophytes affect but differ in the network patterns of archaea and bacterial communities. In general, the greater the stability and complexity of the symbiotic network, the greater the ability to prevent external interference (Lin et al., 2019; Chun et al., 2020), this results also suggest that the loss of submerged macrophytes can have a negative impact on the stability of lake ecosystems.

In shallow grass-type lakes, microbial growth and reproduction are largely dependent on submerged macrophytes. The loss of submerged macrophytes leads to increased organic matter in sediment (Banks and Frost, 2017). It has been found that changes in organic matter can alter microbial co-occurrence networks (Barberán et al., 2012). It is not difficult to understand that organic matter serves as a major nutrient source for bacteria, and the abundance of organic matter in the ML area could be directly ingested and utilized by the bacteria in the sediment, thus reducing the interspecies interactions that sustain their growth and reproduction, and therefore the therefore the connectivity and complexity of the networks are reduced (Chen and Wen, 2021; Hernandez et al., 2021). In water, submerged macrophytes can promote the metabolism of bacteria in the water by releasing fresh substrate. However, with the loss of submerged macrophytes, the metabolism of bacteria in water is inhibited, which leads to decrease in inter-species connectivity (Rimes and Goulder, 1985; Jiao et al., 2020). Thus, the loss of submerged macrophytes reduces the complexity and connectivity of bacterial networks in water. In the archaeal networks, the network stability and complexity of the sediment MA was higher than that of the ML network, most likely also resulting from the availability of sufficient organic matter in the ML area for archaea to utilize, which reduces the inter-species collaboration. In general, species with similar functions cluster together to interact (Jiao et al., 2020). The clusters of *Crenarchaeota* differ in sediment archaeal MA and sediment archaeal ML networks, which may be pH influenced (Yao et al., 2019). The energy metabolism of *Crenarchaeota* was dependent on the pH of the environment (Santos et al., 2021), and submerged macrophyte loss alters sediment acidity and alkalinity, so pH may alter ecological clusters either directly or indirectly (Lauber et al., 2009). Compared to water bacteria, the networks structure of archaea in the water were virtually unaffected by the submerged macrophyte. After all, there are significant differences in cell structure, cell wall component resistance, resistance, and metabolic pathways between archaea and bacteria (Haller et al., 2011), so their interspecific interactions respond to environmental changes to different degrees.

## 5 Conclusion

This study helps to elucidate the effects of submerged macrophytes loss in shallow lakes on the bacterial and archaeal communities in sediment and water. The results showed that the loss of submerged macrophytes had effect on the bacterial and archaeal community compositions in the sediment and the water. More specifically, the loss of submerged macrophytes had significant effect on the bacterial and archaeal community structure in the water, however, it did not have significant effect on those in the sediment. We also verified through RDA analysis that PB was the key factor significantly affecting the bacterial community structure in the water and TN was the factor significantly affecting the archaeal community structure in the water. In addition, the loss of submerged macrophytes had no significant effect on the alpha diversity of bacteria and archaea in the sediment and the water. The co-occurrence network analysis showed that the loss of submerged macrophytes reduced interspecific interactions between bacteria in the sediment and the water. For archaea, the loss of submerged macrophytes reduced the network connectivity and complexity of archaea in the sediment, while the network patterns of archaea in the water did not differ much between the MA network and the ML network. This shows that interactions of bacteria and archaea are differentially effected by loss of submerged macrophytes.

## Data availability statement

The datasets presented in this study can be found in online repositories. The names of the repository/repositories and accession number(s) can be found at: https://www.ncbi.nlm.nih.gov/, PRJNA1067192.

## Author contributions

JL: Conceptualization, Data curation, Formal analysis, Investigation, Methodology, Software, Visualization, Writing – original draft, Writing – review & editing. XH: Investigation, Methodology, Writing – original draft, Writing – review & editing. XJ: Data curation, Writing – review & editing. CQ: Conceptualization, Data curation, Resources, Writing – review & editing. YL: Investigation, Writing – review & editing. PX: Conceptualization, Data curation, Methodology, Writing – review & editing.
