# Supplementary material for: Loss of submerged macrophytes in shallow lakes alters bacterial and archaeal community structures, and reduces their co-occurrence networks connectivity and complexity
Source: Front Microbiol. 2024 Mar 26;15:1380805. doi: 10.3389/fmicb.2024.1380805 (PMC11004660; doi:10.3389/fmicb.2024.1380805)
Supplement: Supplementary file 1 [file Table_1.docx]

Supplementary Material

Loss of Submerged Macrophytes in Shallow Lakes Alters Bacterial and Archaeal Community Structures, and Reduces Their Co-occurrence Networks Connectivity and Complexity

| Author | affiliation | Email |
| --- | --- | --- |
| Jiahui Liu^1^ | Guizhou Province Key Laboratory for Information System of Mountainous Areas and Protection of Ecological Environment, Guizhou Normal University, Guiyang, Guizhou, China | h1052228049@163.com |
| Xianfei Huang^1^ | Guizhou Province Key Laboratory for Information System of Mountainous Areas and Protection of Ecological Environment, Guizhou Normal University, Guiyang, Guizhou, China | hxfswjs@gznu.edu.cn |
| Xin Jiang^1^ | Guizhou Province Key Laboratory for Information System of Mountainous Areas and Protection of Ecological Environment, Guizhou Normal University, Guiyang, Guizhou, China | jiangxin@gznu.edu.cn |
| Chun Qing^1^ | Guizhou Province Key Laboratory for Information System of Mountainous Areas and Protection of Ecological Environment, Guizhou Normal University, Guiyang, Guizhou, China | qingchun@cug.edu.cn |
| Yue Li^2^ | Guizhou Caohai National Nature Reserve Management Committee, Bijie, Guizhou, China | muzifengyue@126.com |
| Pinhua Xia^1^* (corresponding author) | Guizhou Province Key Laboratory for Information System of Mountainous Areas and Protection of Ecological Environment, Guizhou Normal University, Guiyang, Guizhou, China | pinhuaxia@gznu.edu.cn |

*** Correspondence:**Pinhua Xia
[pinhuaxia@gznu.edu.cn](mailto:pinhuaxia@gznu.edu.cn)

# Tables

**Table S1 Alpha diversity index of bacteria and archaea in sediment and water in submerged macrophyte abundant (MA) and submerged macrophyte loss (ML) areas (mean±SD,n=6)**

|  |  |  | shannon | simpson | Chao1 | PD |
| --- | --- | --- | --- | --- | --- | --- |
| Bacteria | Sediment | MA | 5.98±0.45a | 0.02±0.03a | 983.33±166.46a | 98.38±17.50a |
|  |  | ML | 5.94±0.45a | 0.01±0.01a | 953.5±195.98a | 98.6±21.15a |
|  | Water | MA | 5.00±0.35a | 0.02±0.01a | 527.33±132.06a | 56.13±10.68a |
|  |  | ML | 4.92±0.17a | 0.02±0.003a | 437.83±67.11a | 51.76±10.56a |
| Archaea | Sediment | MA | 4.48±0.41a | 0.03±0.02a | 194±39.40a | 8.50±1.70a |
|  |  | ML | 4.35±0.32a | 0.03±0.01a | 192±46.36a | 9.37±2.32a |
|  | Water | MA | 4.48±0.32a | 0.02±0.01a | 207±59.61a | 18.11±3.60a |
|  |  | ML | 4.52±0.23a | 0.03±0.01a | 226.83±29.33a | 25.23±2.23a |

In the same line, the same letter indicates that the difference is not significant, and the different letters indicate that the difference is significant (P < 0. 05). "n" represents the number of sampling points. 6 sampling points in the submerged macrophyte abundant (MA) area (L1~L6; *n*=6); 6 sampling points in the submerged macrophyte loss (ML) area (L7~L12; *n*=6). Abbreviations: SD, Standard Deviation; PD, Phylogenetic Diversity.

**Table S2 Mean value of water chemical parameters and submerged macrophyte characteristics (mean±SD) in the submerged macrophyte abundant (MA) and submerged macrophyte loss (ML) areas**

|  | submerged macrophyte abundant (MA) area (*n=*6) | submerged macrophyte loss (ML) area (*n*=6) |
| --- | --- | --- |
| TP (mg-P/L) | 0.03±0.02a | 0.02±0.01a |
| TN (mg-N/L) | 1.33±0.21a | 1.25±0.04a |
| NH_3-_N (mg/L) | 0.65±0.2a | 0.64±0.2a |
| COD_Mn_ (mg/L) | 7.78±0.47a | 7.58±0.09a |
| Chl *a* (mg/m³) | 11.35±4.22a | 12.09±4.2a |
| DO (mg/L) | 8.0±0.16a | 7.50±0.18b |
| pH | 8.78±0.17a | 8.68±0.16a |
| ORP (mV) | 145.35±14.42a | 133.37±12.90a |
| WT (cm) | 95.33±25.89a | 99.33±14.26a |
| WD (cm) | 117±23.45a | 183±22.67b |
| WT/WD | 0.95±0.03a | 0.88±0.03b |
| PB (g/㎡) | 728.62±527.96a | 12.73±16.53b |
| PC (%) | 43.83±25.78a | 0.83±1.19b |
| species of submerged macrophyte | *Potamogeton pectinatus* L., *Potamogeton malaianus* Miq., *Najas marina* L., *Nitella acuminate* B., and *Myriophyllum spicatum* L. | *Najas marina* L. and *Nitella acuminate* B. |

The data were obtained mean±SD of doth sets of samples; In the same line, the same letter indicates that the difference is not significant, and the different letters indicate that the difference is significant (P < 0. 05). "n" represents the number of sampling points. 6 sampling points in the submerged macrophyte abundant (MA) area (L1~L6; *n*=6); 6 sampling points in the submerged macrophyte loss (ML) area (L7~L12; *n*=6). Abbreviations: SD, Standard Deviation ;TN, total nitrogen; TP, total phosphorus; NH_3_-N, ammonia nitrogen; Chl a, Chlorophyll a; COD_Mn_, chemical oxygen demand; DO, dissolved oxygen; ORP, Oxidation-Reduction Potential; WT, water transparency; WD, water depth; PB, plant biomass; PC, plant cover.

# Figures


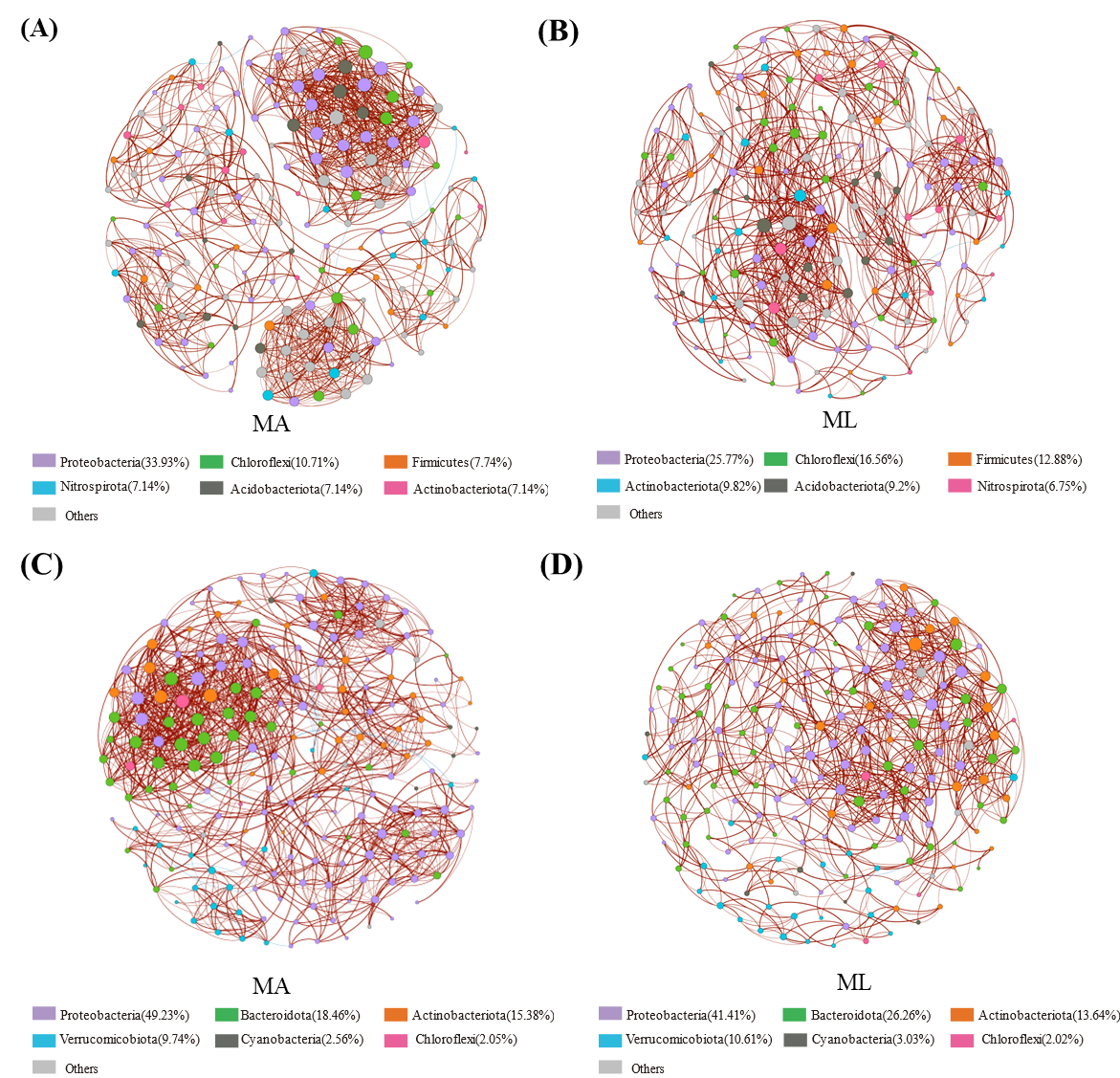


**Figure S1.** Co-occurrence networks of the sediment and water bacteria community in the submerged macrophyte abundant area (MA) and the submerged macrophyte loss (ML) area.The size of each node is proportional to the number of connections (i.e. degrees) and they are colored by classification of species at the phylum level. The red and blue edges (lines) in the network indicate significant positive and negative correlations, respectively. Sediment bacterial networks in the MA **(A)** area and ML **(B)** area; water bacterial networks in the MA **(C)** area and ML **(D)** area.


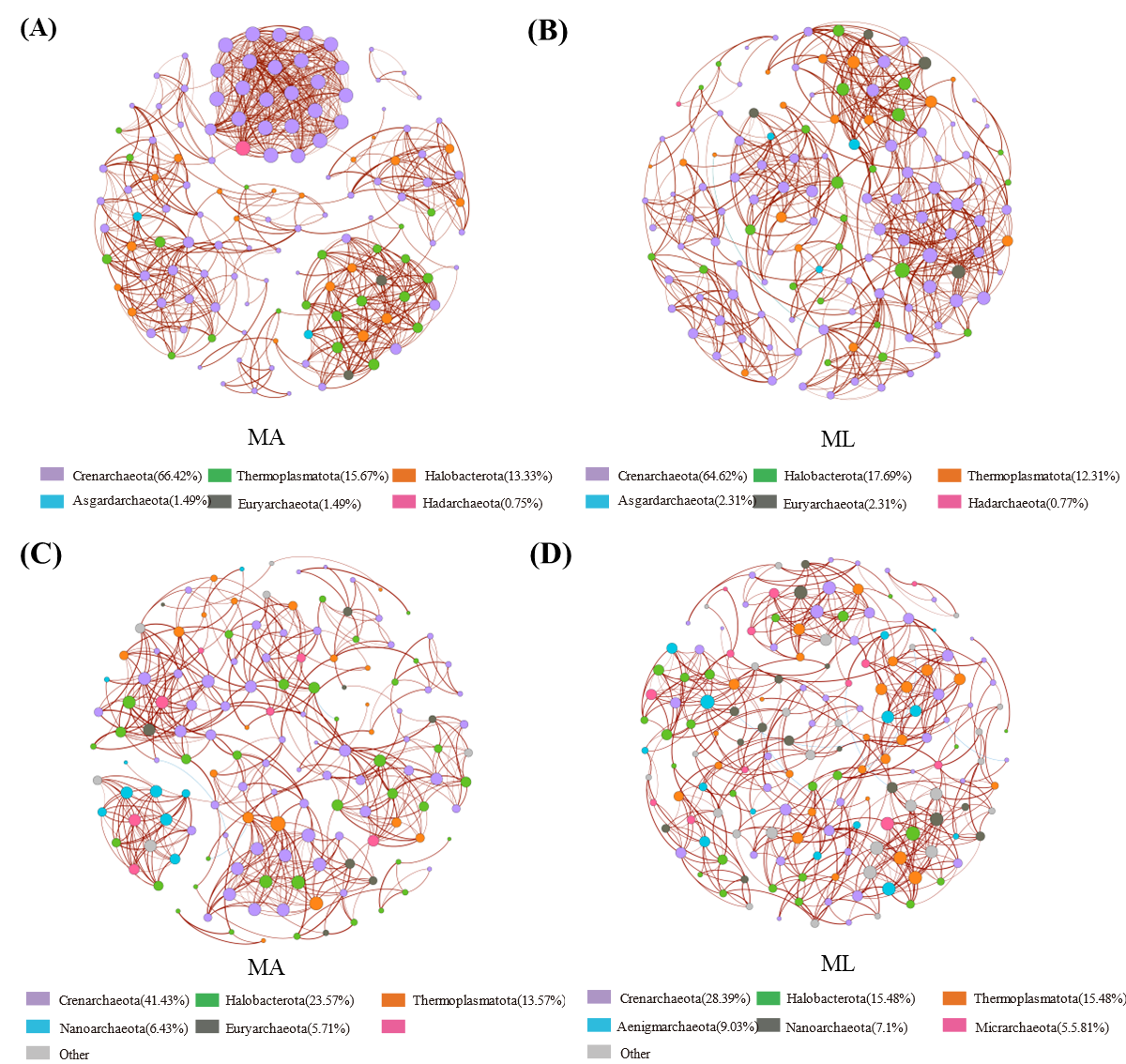


**Figure S2.** Co-occurrence networks of the sediment and y water archaeal community in submerged macrophyte abundant (MA) area and submerged macrophyte loss (ML) area. The size of each node is proportional to the number of connections (i.e. degrees) and they are colored by classification of species at the phylum level. The red and blue edges (lines) in the network indicate significant positive and negative correlations, respectively. Sediment bacterial networks in the MA **(A)** area and ML **(B)** area; water bacterial networks in the MA **(C)** area and ML **(D)** area.
